# Exploration of acute and chronic anti-inflammatory potential of *Quercus leucotrichophora A. Camus* extracts in Wistar rats: A mechanistic insight

**DOI:** 10.3389/fphar.2023.1002999

**Published:** 2023-04-11

**Authors:** Ammara Saleem, Izza Hameed, Muhammad Furqan Akhtar, Ghulam Md Ashraf, Badrah S. Alghamdi, Md. Habibur Rahman, Majed N. Almashjary

**Affiliations:** ^1^ Department of Pharmacology, Faculty of Pharmaceutical Sciences, Government College University Faisalabad, Faisalabad, Pakistan; ^2^ Riphah Institute of Pharmaceutical Sciences, Riphah International University, Lahore Campus, Lahore, Pakistan; ^3^ Department of Medical Laboratory Sciences, College of Health Sciences, Sharjah Institute for Medical Research, University of Sharjah, Sharjah, United Arab Emirates; ^4^ Pre-Clinical Research Unit, King Fahd Medical Research Center, King Abdulaziz University, Jeddah, Saudi Arabia; ^5^ Department of Physiology, Neuroscience Unit, Faculty of Medicine, King Abdulaziz University, Jeddah, Saudi Arabia; ^6^ Department of Pharmacy, Southeast University, Banani, Dhaka, Bangladesh; ^7^ Department of Medical Laboratory Sciences, Faculty of Applied Medical Sciences, King Abdulaziz University, Jeddah, Saudi Arabia; ^8^ Hematology Research Unit, King Fahd Medical Research Center, King Abdulaziz University, Jeddah, Saudi Arabia; ^9^ Animal House Unit, King Fahd Medical Research Center, King Abdulaziz University, Jeddah, Saudi Arabia

**Keywords:** *Quercus leucotrichophora*, oxidative stress biomarkers, complete Freund’s, HPLC, TNF-α, IL-6

## Abstract

**Introduction:** This research was conducted to validate the folkloric use of *Quercus leucotrichophora* (QL) leaf extracts against inflammation and arthritis and to determine the chemical composition using HPLC.

**Method:** The aqueous and methanolic extracts of QL were evaluated by *in vitro* anti-oxidant, anti-inflammatory (inhibition of protein denaturation and membrane stabilization) assays, and *in vivo* anti-inflammatory (carrageenan and xylene-induced edema) and anti-arthritic models. For anti-arthritic potential, 0.1 mL Complete Freund’s Adjuvant (CFA) was inoculated into the left hind paw of a Wistar rat on day 1, and oral dosing with QL methanolic extract (QLME) at 150, 300, and 600 mg/kg was begun at day 8 till the 28th day in all groups, except disease control that was given distilled water, while methotrexate was given as standard treatment.

**Results and discussion:** There was a noteworthy (*p* < 0.05–0.0001) restoration in body weight, paw edema, arthritic index, altered blood parameters, and oxidative stress biomarkers in treated rats as compared to the diseased group. Moreover, QLME treatment significantly (*p* < 0.0001) downregulated TNF-α, IL-6, IL-1β, COX-2, and NF-κB, while significantly (*p* < 0.0001) upregulating IL-10, I-κB, and IL-4 in contrast to the diseased group. The QLME exhibited no mortality in the acute toxicity study. It was concluded that QLME possessed substantial anti-oxidant, anti-inflammatory, and anti-arthritic potential at all dosage levels prominently at 600 mg/kg might be due to the presence of quercetin, gallic, sinapic, and ferulic acids.

## Graphical Abstract

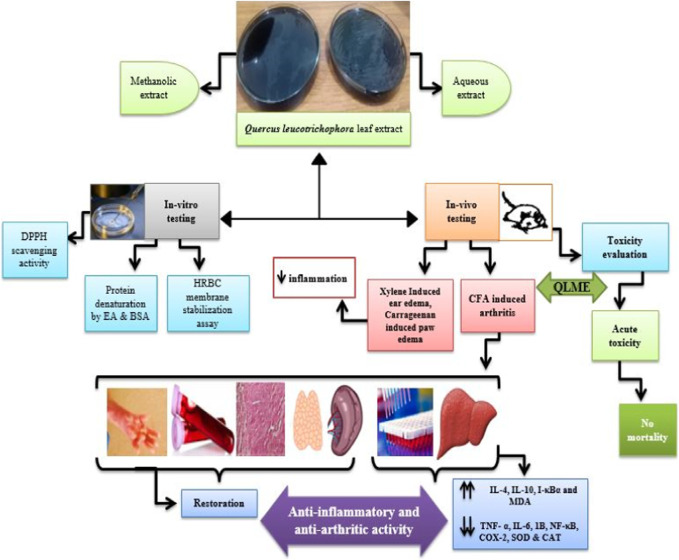

## Introduction

Inflammation is thought to be a body’s non-specific immune response as a result of infection or injury, detecting foreign invaders and triggering an immune response or healing. The process of inflammation can be assessed by major cardinal signs at the tissue level that include tumor (swelling), calor (heat), rubor (redness), dolor (pain), and functio laesa (loss of function) (Stankov, 2012).

Rheumatoid arthritis (RA), a chronic inflammatory auto-immune syndrome of intricate etiology, is symbolized by morning stiffness, fatigue, persistent swelling of joints, erosive synovitis, and joint rigidity that begins predominantly in small diarthrodial joints of hands and feet. It affects 0.5%–1% of the adult population worldwide. Its susceptibility increases with age. The risk factors for RA include diet, smoking, hormones, caffeine, and genetic abnormalities (Firestein, 2003; Oliver and Silman, 2006).

RA is a multifaceted disease clinically identified by the existence of rheumatoid factor (RF) or anti-citrullinated protein antibody in the sera of affected persons. There is also an augmented discharge of pro-inflammatory cytokines, for instance, tumor necrosis factor (TNF)-α, interleukin (IL)-6, 1β, and metabolic enzymes like cyclooxygenase (COX)-2 and lipoxygenases (LOX) from activated immune cells. However, the level of anti-inflammatory cytokines (IL-4 and 10) is decreased in affected individuals (Harrison et al., 2021; Zhang, 2021). Oxidative stress is another trigger factor for RA in addition to pro-inflammatory cytokines.

RA management includes medications, physiotherapy, counseling of patients, nutrition, and surgery. The medications include non-steroidal anti-inflammatory drugs NSAIDs (diclofenac, ibuprofen, and piroxicam); glucocorticoids (prednisolone and dexamethasone); biologics; anti-TNF-α; gold compounds; and disease-modifying anti-rheumatic drugs (DMARDs) (Placha and Jampilek, 2021). As RA is not curable, its lifelong therapy is expensive and complicated, with various adverse effects such as ulceration, bone marrow depression, cardiovascular disorders, hypertension, hepatic damage, and nephrotoxicity. The practice of using medicinal plants such as *Nigella sativa*, *Moringa oleifera, Polystichum braunii*, and *Caralluma tuberculata* for treating various diseases, for instance, arthritis, asthma, diabetes, and microbial infections, provides a preferable alternative due to its low cost, ease of access, and minimum harmful implications (Shabbir et al., 2018).

*Quercus leucotrichophora* A. Camus (QL) belongs to the Fagaceae family. It is a deciduous evergreen tree and is commonly called “Rein”. The genus *Quercus* includes about 450 species. QL is widespread in Asia, Europe, North Africa, and Central and South America, among other regions with trees of this genus. QL grows in temperate and tropical climatic regions and is used as an indigenous medicine for RA in the Rawalpindi district, Pakistan. Over the centuries, the various species of the genus *Quercus* (*Quercus dilatata* and *Quercus incana*) have been traditionally utilized for treatment of pain, inflammation, and arthritis (Moreno-Jimenez et al., 2015); (Taib et al., 2020).

The plant (leaves, bark, and wood) is used as a folkloric remedy to treat rheumatism, asthma, dysentery, diuretic, backache, cough, fever, and joint pain (Saqib et al., 2014). The leaf, seed, and bark of QL are used for therapeutic purposes for livestock also. Its bark and leaves contained 23 and 62 constituents, respectively, with profound anti-bacterial activity (Semwal et al., 2018). It has significant antioxidant and hepato-protective activity (Singh and Bisht, 2018). The literature survey showed that the anti-arthritic potential of QL was not scientifically validated. Therefore, the current study evaluated the acute and chronic anti-inflammatory potential of QL extracts using *in vitro* and *in vivo* methods. Moreover, acute toxicity testing and chemical characterization of the plant extracts were also investigated.

## Materials and methods

### Sample preparation

QL leaves from a fully grown tree ( 20 m) were collected in November 2020 from Murree, Pakistan, and were identified by a taxonomist at the University of Agriculture, Faisalabad (Voucher No. 1117-20-5b), and the sample was submitted for referencing.

The leaves (5 kg) were washed to eliminate dust and foreign particles, then dried under shade, and ground to form a coarse powder. The methanol and aqueous extracts of QL (QLME and QLAQ) were prepared by maceration. The powdered plant was soaked in methanol (1:10) for 14 days at 37 °C. Filtration was carried out using a muslin cloth followed by Whatman No. 1 filter paper, and the steps were repeated twice. Finally, filtrates were gathered and condensed using a rotary evaporator at 40°C at 100 mbar. It was then air-dried at room temperature until completely dried. The same process was repeated for preparing the QLAQ extract, while QLAQ was condensed in the rotary evaporator at 90°C (Nagappan, 2012; Abubakar and Haque, 2020).

### Animals for experimentation

Healthy Wistar rats of both sexes (120–170 g; age 12–15 weeks) were obtained and kept at the Animal House of the Department of Pharmacology, Government College University, Faisalabad with a continuous supply of water and a standard pellet diet. The animals were kept in 12 h of light/dark cycles at standard humidity (60%–70%) and temperature (28°C–30°C). The approval for animal testing was acquired from The Ethical Committee of Animals, GCUF (Voucher # GCUF/ERC/2220). Undue damage to the animals was evaded.

### Qualitative phytochemical analysis of extracts

The QL extracts were evaluated for presence of phytochemicals like alkaloids, tannins, phenols, saponins, flavonoids, steroids, terpenoids, proteins, and glycosides by standard procedures (Saleem et al., 2020a).

For determining total phenolic contents (TPCs), the extract (0.5 mL) was mixed with 2.2 mL of distilled water (DW) and 0.15 mL of 5% NaNO_2_ solution. Then, after 6 min, 0.3 mL of 10% AlCl_3_.6H_2_O was mixed and left for 5 min. Then, 1 mL of 1N NaOH solution was added and vortexed; later, the absorbance was measured at 510 nm. Gallic acid was used as the standard (Saleem et al., 2020b).

### Quantitative phytochemical analysis of extracts

For total flavonoid contents (TFCs), 100 µL of the stock sample (10 mg/mL) was added to 2 mL (2% Na_2_CO_3_) and left for 2 min at room temperature. Afterward, 100 µL of 50% Folin–Ciocalteu’s reagent was added. Catechin was used as the reference. After 30 min of incubation at room temperature, absorbance was determined at 765 nm (Cheruth et al., 2016).

The HPLC analysis of QLME and QLAQ was performed by following the previously mentioned procedure (Saleem et al., 2020a). The sample preparation was performed by adding 10 mg of the extract to 5 mL of DW, and then, 12 mL of ethanol was mixed. After 5 min, 6 mL DW was added and held for 5 min, along with the addition of 10 mL of 15 M HCl, and then placed in the oven for 2 h at 90°C. A syringe filter was used for injecting the sample into HPLC. The separation of compounds was conducted using the Shim–Pack Column (Shimadzu, Japan) CLC-ODS. The mobile phase included methanol and acetonitrile in a ratio of 30:70 as solvent A and double-DW with glacial acetic acid (0.5%) as solvent B. The UV–Visible detector (SPD-10AV) was used at a wavelength of 280 nm. The retention time of standards was used to identify and quantify detected compounds.

#### *In vitro* evaluation

##### i. 2,2-Diphenyl-1-picrylhydrazyl (DPPH) scavenging assay

For determining antioxidant potential, 2 mL of DPPH solution (0.04/100 mg methanol) was mixed with 1 mL solution of plant extract and 1 mL of methanol. The two-fold dilution method was used to prepare the sample solution of extracts in methanol. Absorbance was measured after 30 min at 517 nm using ascorbic acid as a reference (Saleem et al., 2020a). The % DPPH radical scavenging of the mean was calculated.

##### ii. Inhibition of protein denaturation assays

In the egg albumin (EA) denaturation assay, a 5 mL reaction mixture containing 0.2 mL of the EA (from a fresh egg of a hen); phosphate-buffered saline 2.8 mL (PBS) of pH 6.4; and 2 mL of plant extracts (50, 100, 200, 400, 800, and 1,600 μg/mL) was used, while the same volumes of piroxicam solution and DW were used as standard and control solutions, respectively, in place of extract solutions. Afterward, these mixtures were incubated for 15 min at 37 ± 2°C. Later, they were warmed for 5 min at 70°C. The absorbance of the mixture was measured at 660 nm (Akhtar, 2020).

For the BSA denaturation assay**,** a previously implemented procedure was followed (Saleem et al., 2019). Briefly, the test control (0.5 mL) contained 0.45 mL of BSA (5% w/v) and 0.05 mL of extract dilutions. The product control solution contained DW (0.45 mL) in place of the BSA solution. The standard solution contained piroxicam instead of extract solution. The pH was adjusted to 6.3, incubated for 20 min at 37°֯C, and afterward, heated at 57°C for 3 min. The absorbance was measured at 660 nm.

##### iii. Human red blood cell (HRBC) membrane stabilization assay

This assay was performed according to a previous procedure (Saleem et al., 2019). In brief, 3 mL of blood from a healthy volunteer was mixed with Alsever’s solution, and RBC suspension (10% v/v) was prepared using an isosaline solution after centrifugation at 3,000 rpm for 15 min.

The test solution contained phosphate buffer (1 mL), hypotonic saline (2 mL), 0.5 mL of extract, and 10% human red blood cells (0.5 mL)**.** The test control solution contained DW, while the standard solution contained piroxicam instead of the extract. The solutions were incubated for 30 min at 37°C and centrifuged at 3,000 rpm for 15 min. The sample absorbance was measured at 560 nm, and % protection was calculated.

#### Acute toxicity study

It was performed by following OECD guidelines 425 up-and-down procedure. A total of 10 female rats (120–170 g) were distributed into two groups (n = 5). Briefly, l00 mg/kg dose of QLME was given and change in behavior, gait, movements, respiration, and mortality was observed at 0.5, 1, 2, and 4 h till the 48th hour. While in NC, only 1 mL of DW was given. The rats were observed for clinical signs of toxicity like mortality, body temperature, respiratory rate, and motor movements during this period. The body weight was also measured on 1, 7, and 14th days (Saleem et al., 2020b).

#### Acute anti-inflammatory potential in rats

i- Carrageenan-induced paw edema

The 54 Wistar rats (120–170 g) were randomly allocated into six groups (six rats in each group): Group I served as normal control (NC) provided with DW; Group II as disease control (DC) provided with DW; Group III as standard control (SC) receiving piroxicam (10 mg/kg); and Groups IV, V and VI were given QLME at 150, 300, and 600 mg/kg and QLAQ at 150, 300, and 600 mg/kg orally, respectively.

The rats in all groups were treated as aforementioned. One hour later, 0.1 mL of carrageenan (1% w/v) solution was administered *via* subcutaneous injection in the left hind paw (sub-plantar region) of all rats, except NC. Paw diameter (mm) was measured at 0, 1, 2, 3, and 4 h till the 8^th^ hour using a digital Vernier caliper (Shabbir et al., 2018).

#### ii- Xylene-induced ear edema

A previous procedure was adopted for xylene-induced ear edema (Shabbir et al., 2018). Briefly, a drop of xylene was applied to the inner surface of the right ear of each animal except NC, 60 min post-administration of the previously mentioned treatments. After that, the anesthetized rats were euthanized after 15 min to remove and weigh both ears. The percentage inhibition was calculated.

$$Increase\;in\;weight=right\;ear\;weight–left\;ear\;weight;\;\%\;inhibition=\left(C-T\right)/\left(C\right)\times 100.$$

C: disease control; T: treatment.

### Anti-arthritic potential in rats

#### Complete Freund’s Adjuvant- induced arthritis

The 36 Wistar rats (120–170 g) were indiscriminately allocated into six groups (n = 6). On day 1, 0.1 mL CFA (Sigma Aldrich^®^, United Kingdom) was injected in the left hind paw in all, excluding normal control rats (Han et al., 2016).The treatment was started from day 8 after inoculation till 28th day, and the treatment procedure is as follows. Group I served as normal control (NC) and was provided with DW. Group II was the disease control (DC) and was provided with DW. Group III was standard control (SC) and was given methotrexate (MTX) intraperitoneally. Groups IV, V, and VI were treated with QLME at 150, 300, and 600 mg/kg *via* orally, respectively, for 21 days.

Treatment plan

**Table:** 

Groups	Treatment
I (normal control)	Distilled water; 1 mL
1I (disease control)	RA + distilled water; 1 mL
111 (standard control)	RA + methotrexate (1 mg/kg/week)
IV	RA + QLME 150 mg/kg; 1 mL
V	RA + QLME 300 mg/kg; 1 mL
VI	RA + QLME 600 mg/kg; 1 mL

### Evaluation of arthritis

The body weight and diameter of the left hind paw were determined before the first immunization and then on days 7, 12, 16, 20, 24, and 28th. The percentage inhibition of paw edema was measured. The severity of arthritis was assessed by an arthritic scoring system that ranged from 0 to 4 scales. The arthritic scoring included 0: no swelling; 1: swelling of toe joints; 2: swelling of both toes and toe joints; 3: ankle joint swelling; and 4: entire paw swelling leading to immobility (Han et al., 2016).

### Effect on blood parameters

After the 28th day, the blood was collected via cardiac puncture in collecting tubes from anesthetized rats. The commercially available kit (Antec Diagnostic Products^®^, United Kingdom) was utilized to determine RF, alanine aminotransferase (ALT), aspartate aminotransferase (AST), and alkaline phosphatase (ALP), while an automated chemistry analyzer (Microlab 300^®^, Germany) was used to determine urea and creatinine. The automated hemocytometer (Sysmex, Roche^®^, Germany) was used to count complete blood count (CBC).

### Effect on immune organs and histopathology of ankle joint

The blood collected by cardiac puncture in the EDTA tube was processed for RNA extraction. The abdomen was dissected to remove the spleen and thymus, which were then washed with DW and weighed (Saleem et al., 2020c). For histopathological evaluation, left hind limb ankle joints were removed, rinsed with DW, and placed for 24 h in 10% v/v neutral-buffered formalin. The decalcification of joints was performed by decalcifying solution (10% w/v EDTA). After mounting on slides, the tissues were stained, and the slides were observed for histopathological changes under a light microscope at 40X (Shabbir et al., 2018).

### Quantification of inflammatory biomarkers by real-time (RT)-PCR

RT-PCR was used for estimating the levels of IL-4, IL-10, IL-6, IL-1β, NF-κB, TNF-α, I- ΚB, and COX-2 in the blood of rats. The RNA was removed from the collected blood by the TRIzol method as per the manufacturer’s instructions (Invitrogen®Pure Link RNA). The complementary DNA synthesis was performed using the kit manufacturer’s protocol (K1622: Thermo Scientific^®^, Germany). For quantification and amplification, the SYBR Select Master Mix kit (Applied Biosystems Thermo Scientific^®^, Germany) was used on qRT-PCR (Applied Biosystems Thermo Scientific^®^). The primers were selected from a previous study using GAPDH as the housekeeping gene (Saleem et al., 2020d).

### Assessment of oxidative stress biomarkers

After the 28th day, rats were euthanized, and the liver was taken out for estimating superoxide dismutase (SOD), catalase (CAT) , and malondialdehyde (MDA) levels. The 10% liver homogenate (LH) was prepared (Akhtar et al., 2016). Lowry’s method was used for estimating protein content in LH (Saleem et al., 2021). The SOD, CAT, and MDA in the LH were estimated by xanthine oxidase, hydrogen peroxide, and thiobarbituric acid (TBA) assays, respectively (Bhangale and Acharya, 2016).

### Statistical analysis

The data were expressed as mean ± standard deviation (S.D). *In vitro* assays, xylene-induced ear edema, and blood results were analyzed using one-way analysis of variance (ANOVA) followed by Tukey’s test and others by two-way ANOVA followed by Tukey’ s test using GraphPad Prism^®^ software version 7.0. *p* < 0.05 was considered statistically significant.

## Results

The percentage yields of QLME and QLAQ were 6.1% and 4.05%, respectively.

### Phytochemical analysis

It was observed that tannins, phenols, glycosides, terpenoids, and flavonoids were present in QLME and QLAQ, while alkaloids and carbohydrates were present only in QLAQ. Saponins, proteins, and steroids were absent in both extracts. QLME contained a higher amount of phenolic acid and flavonoids (TPC: 201.2 ± 0.72 mg GAE/g and TFC: 30.30 ± 0.66 mg CE/g) than QLAQ (TPC:188.07 ± 0.50 mg GAE/g and TFC: 23.37 ± 0.85 mg CE/g).

### Quantitative analysis

The HPLC analysis revealed that QLME contained the highest amount of quercetin (265.53 ppm) followed by gallic acid (190.0389 ppm). The QLAQ had the highest amount of p-coumaric acid (255.34 ppm) followed by catechin (236.9 ppm). The phenolic acid and flavonoids detected in the plant extracts are listed in Table 1.

**TABLE 1:** HPLC profile of phytochemicals detected in methanolic and aqueous extracts of *Quercus leucotrichophora*.

Extracts	Compound name	Retention time (min)	Concentration (ppm)
*Q. leucotricophora* methanolic extract	Caffeic acid	8.05	7.71
	Gallic acid	3.12	190.04
	Sinapic acid	12.53	73.86
	Ferulic acid	12.76	25.28
	Quercetin	25.02	265.53
	Hydroxy benoic acid	7.00	9.25
*Q. leucotricophora* aqueous extract	Catechin	3.27	236.90
	P-coumaric acid	5.58	255.34
	Ferulic acid	12.83	9.32
	Gallic acid	3.49	23.19
	Sinapic acid	12.23	38.57
	Chlorogenic acid	5.24	12.62
	Caffeic acid	7.30	27.47
	Vanillic acid	8.35	59.38

### *In vitro* antioxidant activity

**FIGURE 1:**
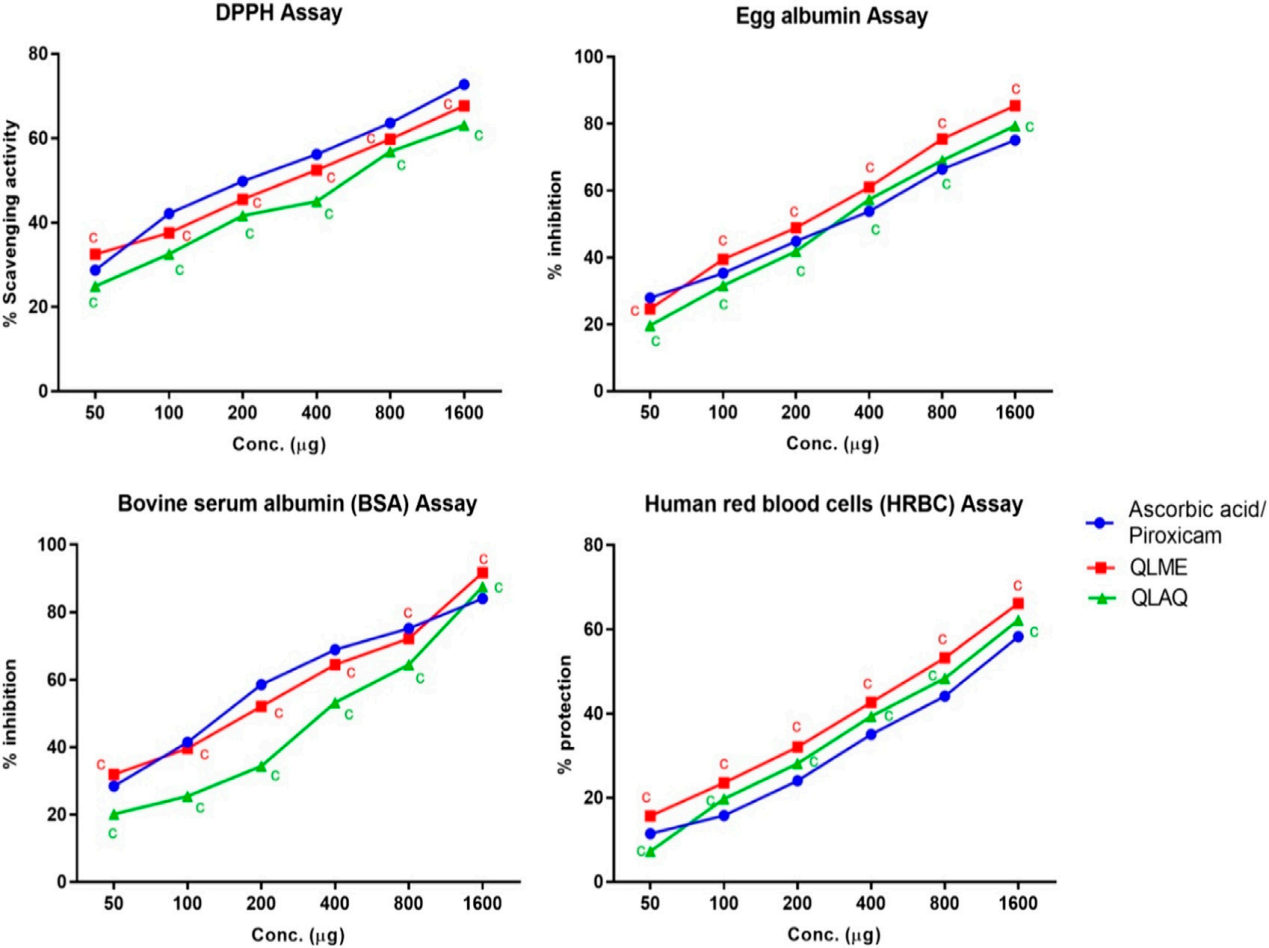
*In vitro* antioxidant (DPPH assay) and *in vitro* anti-inflammatory (protein denaturation and HRBC membrane stabilization assays) activities of *Quercus leucotrichophora* extracts. Results presented as mean ± S.D (n = 3). ‘c’ (*p* < 0.0001) in contrast to standard control. QLME: *Q. leucotrichophora* methanolic extracts*;* QLAQ: *Q. leucotrichophora* aqueous extracts.

The DPPH assay revealed that both extracts exhibited profound dose-dependent antioxidant activity. The highest % scavenging was presented by the QLME 1600 µg/mL solution than the QLAQ 1600 µg/mL solution. The antioxidant activity of QLME (67.64% ± 0.43%) and QLAQ (63.09% ± 0.65%) at 1,600 µg/mL was significantly (*p* < 0.0001) different as compared to that of ascorbic acid (72.75% ± 0.21%), as shown in Figure 1. The QLME exhibited significant (*p* < 0.05) antioxidant activity at all tested concentrations (50, 100, 200, 400, 800, and 1,600 µg/mL) in contrast to QLAQ at the respective concentration.

### Inhibition of protein denaturation

The EA and BSA denaturation inhibition assays revealed that both extracts inhibited protein denaturation at all concentrations. In the egg albumin assay, the maximum % inhibition was shown by QLME (85.42% ± 0.42%) than QLAQ (79.44% ± 0.52%) at 1,600 µg/mL, and both were significantly different as compared to piroxicam (75.13% ± 0.81%). The QLME profoundly (*p* < 0.0001) inhibited EA denaturation at all concentrations in contrast to QLAQ at respective concentrations. In the BSA assay, the maximum % per inhibition exerted by QLME (87.54% ± 0.73%) and QLAQ (91.74% ± 0.44%) at 1,600 µg/mL was considerably different from that of piroxicam (84.05% ± 0.54%), as displayed in Figure 1. The QLME notably inhibited (*p* < 0.0001) BSA denaturation in contrast to QLAQ at respective concentrations.

### HRBC membrane stabilization assay

It was revealed that both extracts dose-dependently inhibited membrane lysis in RBCs (Figure 1). The increased % protection of RBC’s membrane lysis by QLME (66.16% ± 0.83%) and QLAQ (62.17% ± 0.32%) at 1,600 µg/mL was pointedly different (*p* < 0.0001) in comparison to that of piroxicam (58.23% ± 0.22%). The membrane stabilization activity of QLME was notably (*p* < 0.0001) higher than that of QLAQ.

### Acute toxicity study

After administering 2000 mg/kg QLME, no marked changes in behavioral and physiological parameters were observed. The QLME LD_50_ was more than 2000 mg/kg as mortality was not noticed within 14 days of extract administration. There were non-significant (*p* > 0.05) changes in body weight of the treated group (140.3 ± 1.53 g) as compared to the NC group (139.3 ± 2.52 g) on the 14th day.

### Effect on carrageenan-induced paw edema

The carrageenan administration produced a considerable (*p* < 0.0001) increase in paw diameter in DC rats as compared to NC and standard control from 2^nd^ hr till 8^hr^ hr. All treated groups showed significant (*p* < 0.05–0.0001) reduction in paw edema in comparison with DC and NC at respective time intervals (Table 2). The maximum reduction in paw edema exhibited by QLME (3.59 ± 0.08 mm) and QLAQ (3.77 ± 0.09) at 600 mg/kg was notably (*p* < 0.0001) different as compared to that of piroxicam (3.72 ± 0.49 mm). The paw diameter in all treated groups was insignificantly different from the standard control on 3–8 h except QLME 150 mg/kg. The maximum % inhibition was exhibited by QLME (29.03%) at 600 mg/kg, which was more than that of piroxicam and other groups as given in Table 2. The effect of QLME (150, 300, and 600 mg/kg) and QLAQ (150, 300, and 600 mg/kg) on reduction of paw edema was non-significant (*p* > 0.05) at each hour.

**TABLE 2:** Effect of *Quercus leucotrichophora* extracts in carrageenan-induced paw edema in rats as a function of time and administered doses.

Time	Disease control	Standard control (10 mg/kg)	*Q. leucotricophora* methanolic extract (mg/kg)			*Q. leucotricophora* aqueous extract (mg/kg)		
			150	300	600	150	300	600
0	3.64 ± 0.11	3.66 ± 0.05	3.74 ± 0.09	3.73 ± 0.11	3.57 ± 0.09	3.63 ± 0.13	3.73 ± 0.09	3.68 ± 0.03
1	4.82 ± 0.07	4.60 ± 0.11 (4.49%)	4.72 ± 0.15 (2.01%)	4.74 ± 0.15 (1.52%)	4.64 ± 0.12 (3.67%)	4.74 ± 0.08 (1.66%)	4.69 ± 0.15 (2.56%)	4.67 ± 0.08 (3.04%)
2	5.84 ± 0.09	5.31 ± 0.09 ^**a**^ (9.02%)	5.52 ± 0.10^**a**^ (5.42%)	5.41 ± 0.22 ^**a**^ (7.31%)	5.25 ± 0.13 ^**a**^ (10.05%)	5.66 ± 0.10^a^ (3.03%)	5.53 ± 0.10 ^ac^ (5.25%)	5.39 ± 0.14^a^ (7.59%)
3	5.87 ± 0.09	5.14 ± 0.21 ^**a**^ (12.49%)	5.33 ± 0.13^**a**^ **(**9.25%)	5.19 ± 0.16 ^**a**^ (11.69%)	4.99 ± 0.11 ^**a**^ (15.04%)	5.49 ± 0.09^ac^ (6.41%)	5.33 ± 0.08^a^ **(**9.14%)	5.14 ± 0.17^a^ (12.54%)
4	5.45 ± 0.43	4.61 ± 0.16 ^**a**^ (15.47%)	4.79 ± 0.20^**a**^ (12.17%)	4.68 ± 0.09 ^**a**^ (14.13%)	4.45 ± 0.08 ^**a**^ (18.29%)	4.94 ± 0.20^a^ (9.36%)	4.78 ± 0.06^a^ (12.29%)	4.62 ± 0.16^a^ (15.29%)
5	5.32 ± 0.46	4.36 ± 0.19 ^**a**^ (18.05%)	4.52 ± 0.26^**a**^ (15.10%)	4.39 ± 0.11 ^**a**^ (17.48%)	4.14 ± 0.07 ^**a**^ (22.12%)	4.67 ± 0.10^a^ (12.22%)	4.52 ± 0.11^a^ (15.10%)	4.29 ± 0.03^a^ (19.23%)
6	5.25 ± 0.42	4.07 ± 0.13 ^**a**^ (22.49%)	4.33 ± 0.20^**ac**^ (17.47%)	4.06 ± 0.05 ^**a**^ (22.49%)	3.88 ± 0.05 ^**a**^ (26.05%)	4.45 ± 0.20^a^ (15.18%)	4.29 ± 0.07^a^ (18.11%)	4.03 ± 0.10^a^ (23.13%)
8	5.06 ± 0.54	3.72 ± 0.09 ^**a**^ (26.53%)	4.03 ± 0.10^**a**^ (20.41%)	3.81 ± 0.09 ^**a**^ (24.75%)	3.59 ± 0.08 ^**a**^ (29.03%)	4.13 ± 0.15^a^ (18.37%)	3.99 ± 0.10^a^ (21.19%)	3.77 ± 0.09^a^ (25.41%)

Results are presented as mean ± S.D. (n = 6); two-way ANOVA; Tukey’s test; a: disease control; and c: standard control.

### Effect on xylene-induced ear edema

**FIGURE 2:**
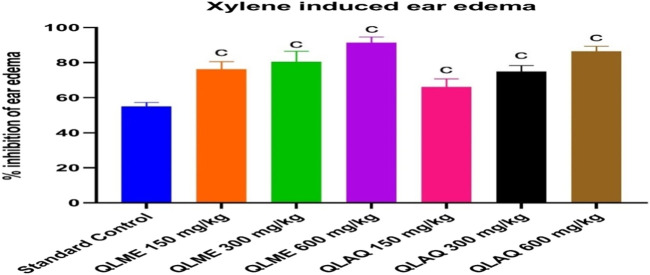
Effect of *Quercus leucotrichophora* extracts in xylene-induced ear edema in rats. Results presented as mean ± S.D. (n = 6); c. *p* < 0.001 as compared to standard control (10 mg/kg). QLME: *Q. leucotricophora* methanolic extract; QLAE: *Q. leucotricophora* aqueous extract. In the previously mentioned *in vitro* testing, QLME was more effective than QLAQ; therefore, QLME was selected for further study to reveal its therapeutic value in treating arthritis.

There was a considerable increase in the ear weight of DC in comparison to NC. The QLME (91.37% ± 3.24%) and QLAQ (86.42% ± 2.88%) significantly (*p* < 0.001) reduced ear edema at 600 mg/kg as equated to the standard control (54.94% ± 2.31%), as presented in Figure 2. The % age inhibitions in QLME and QLAQ 600 mg/kg were notably different from those in QLME and QLAQ 150 and 300 mg/kg treated groups. The QLME at 300 and 600 mg/kg insignificantly (*p* > 0.05) inhibited ear edema in contrast to the corresponding dosage of QLAQ, while QLME 150 mg/kg showed a significant (*p* < 0.05) effect than QLAQ 150 mg/kg in reducing ear edema.

### Effect on paw diameter of arthritic rats

**FIGURE 3:**
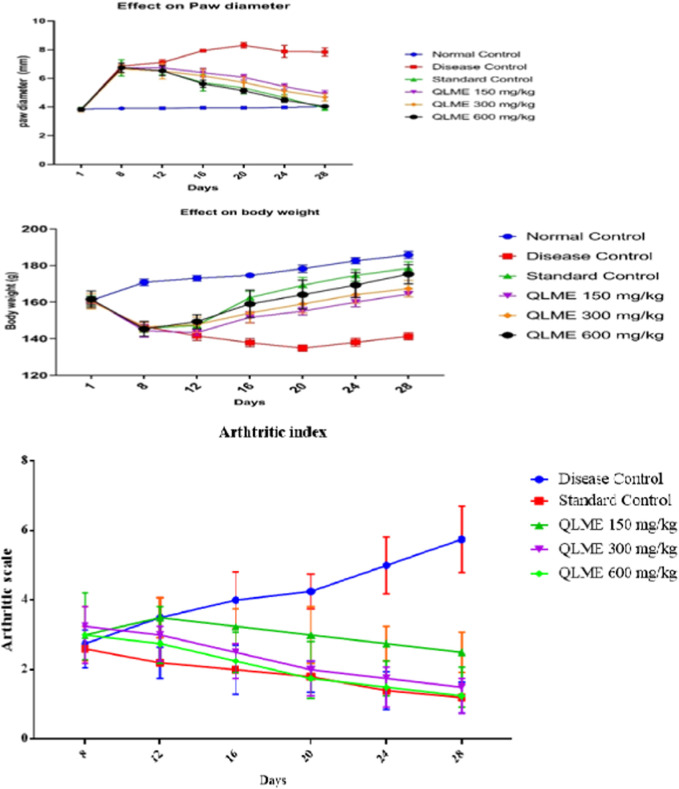
Effect of *Quercus leucotrichophora* extracts on paw diameter, body weight (g), and arthritic index in CFA-induced arthritic rats. Results as mean ± S.D. (n = 6). Analyzed by two-way ANOVA followed by Tukey’s test. QLME: *Quercus leucotricophora* methanolic extract.

The anti-arthritic effect of QLME was evaluated against CFA-induced arthritis in rats because it exhibited notable *in vitro* antioxidant and anti-inflammatory activities. On day 8, there was a considerable increase in paw diameter in all arthritic rats in contrast to NC. A substantial increase in paw diameter in DC rats was noted until the 28th day as compared to NC and standard control groups. The paw diameter in treated groups was notably different from that in NC and DC groups, while QLME-treated groups were insignificantly different from standard control groups on the 12th day (Figure 3). The dosage of 600 mg/kg QLME (4.05 ± 0.07 mm) considerably (*p* < 0.05) reduced paw edema along with MTX-treated rats (3.92 ± 0.16 mm) from 16 to 28th day in contrast to the NC and DC groups. The reduction in paw diameter in standard control rats insignificantly varied from the NC group on the 28th day. The maximum % inhibition was exhibited by MTX (50.02%) followed by QLME 600 mg/kg/day-treated rats (48.34%) on the 28th day, as shown in Table 3. The inhibitory effect of QLME 600 mg/kg on paw edema was insignificantly different from that of MTX on 16 to 28th day (Figure 3).

**TABLE 3:** Effect of *Quercus leucotrichophora* extract on % reduction of paw edema in CFA-induced arthritic rats.

Days	Standard control (1 mg/kg) (%)	*Q. leucotricophora* methanolic extract (mg/kg)		
		150 (%)	300 (%)	600 (%)
12	9.01	5.33	8.99	8.15
16	28.16	19.44	22.16	29.22
20	35.77	26.79	31.25	38.08
24	41.04	31.05	35.19	43.12
28	50.02	37.08	40.43	48.34

### Effect on body weight of arthritic rats

There was a significant decrease in body weight after CFA inoculation in all groups compared to NC on the eighth day. The decrease in weight was continuous in DC from the eighth day as compared to NC till the end of the study. Body weight in all treatment groups was insignificantly different from DC and standard control groups, while significantly (*p* < 0.0001) varying from NC on the 12th day. Body weight in all treatment groups was notably different from NC and DC groups on 16th and 20th days of the study.

However, treatment with QLME (150–600 mg/kg) and MTX caused a significant (*p* < 0.05) restoration of body weight from the 16 to 28th day as compared to DC and NC. Body weight in all treatment groups was notably different from NC and DC groups on 16th and 20th days of the study. Body weight in QLME 600 mg/kg-treated rats was insignificantly different from that in the standard control group on the 20th day. On the 24th day, body weight in all treated groups was significantly different from that of DC and NC, while standard control insignificantly varied from NC and QLME 600 mg/kg. On the 28th day, body weight in all treated groups was notably varied from DC, NC, and standard control groups (Figure 3).

The MTX (178.4 ± 3.65 g)- and QLME 600 mg/kg/day (175.3 ± 5.19 g)-treated rats exhibited considerable (*p* < 0.05) restoration in body weight on the 28th day, in contrast to DC, while the effect of QLME 600 mg/kg/day on body weight was insignificantly varied from MTX on the respective day, as given in Figure 3.

### Effect on arthritic index

During the whole study, the DC group showed continuous increase in arthritic index. The treatment groups exhibited substantial (*p* < 0.05) restoration of the arthritic index from day 16 till the end of the study in contrast to the DC group. The maximum arthritic index was observed in the DC group on the 28th day (4.25 ± 0.96). The plant extract exerted the most pronounced effect on the 28th day in contrast to DC and standard control groups (Figure 3).

### Effect on blood parameters in arthritic rats

**FIGURE 4:**
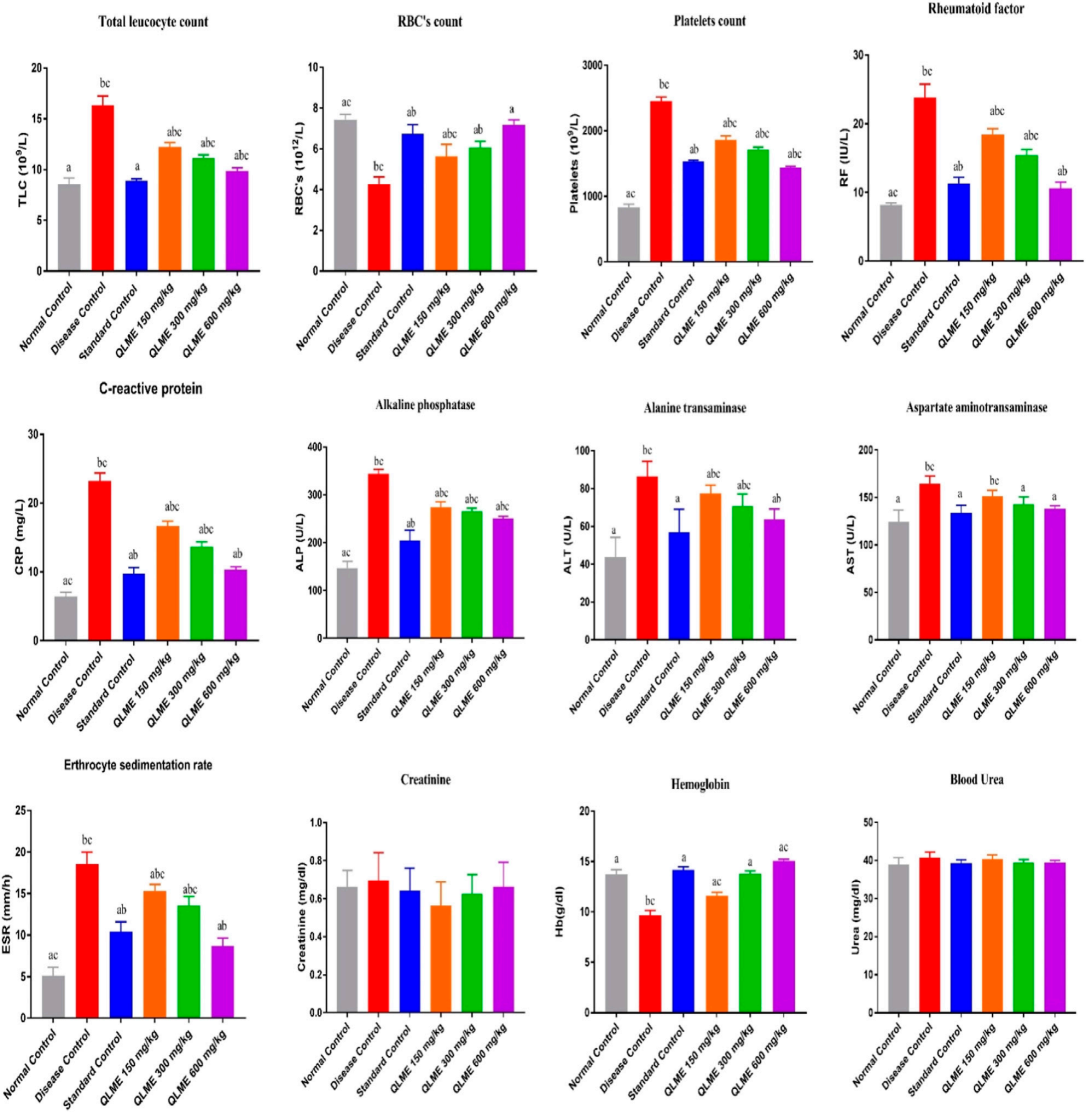
Effects of *Quercus leucotrichophora* extracts on blood parameters in CFA-induced arthritic rats. Results are expressed as mean ± S.D (n = 6) and one-way ANOVA followed by Tukey’s test. a: disease control; b: normal control; and c: standard control. QLME: *Quercus leucotricophora* methanolic extract.

There was a remarkable (*p* < 0.0001) decrease in the level of hemoglobin (Hb) and RBCs, while increase (*p* < 0.05) in platelets, C-reactive protein (CRP), erythrocyte sedimentation rate (ESR), RF, and total leukocyte count (TLC) in DC as compared to NC and standard control groups was observed (Figure 4). Treatment with plant extract (150–600 mg/kg) notably restored Hb and RBCs in treated groups in contrast to NC and DC groups. TLC and Hb in standard control were insignificantly varied from NC. The ESR, CRP, and RF in QLME 600 mg/kg-treated group was insignificantly varied from those of standard control. Moreover, all the treatment groups at 150–600 mg/kg restored the hematological parameters in arthritic rats, as shown in Figure 4.

There were non-significant changes on urea and creatinine levels in all arthritic rats in the 28-day study of CFA induced arthritis. The levels of ALT, ALP and AST were considerably (*p* < 0.05) altered in DC as compared to those of NC and standard control, and all the treatment groups significantly restored their level in arthritic rats profoundly varied from DC and NC, except AST level in QLME 150 mg/kg, which was non-significantly varied from DC (Figure 4). The level of AST in QLME 300- and 600 mg/kg-treated groups was insignificantly varied from standard control. ALP in QLME-treated groups was significantly varied from that of standard control. The ALT in the QLME 600 mg/kg-treated group was insignificantly varied from that of standard control.

### Effect on weight of spleen and thymus in arthritic rats

**FIGURE 5:**
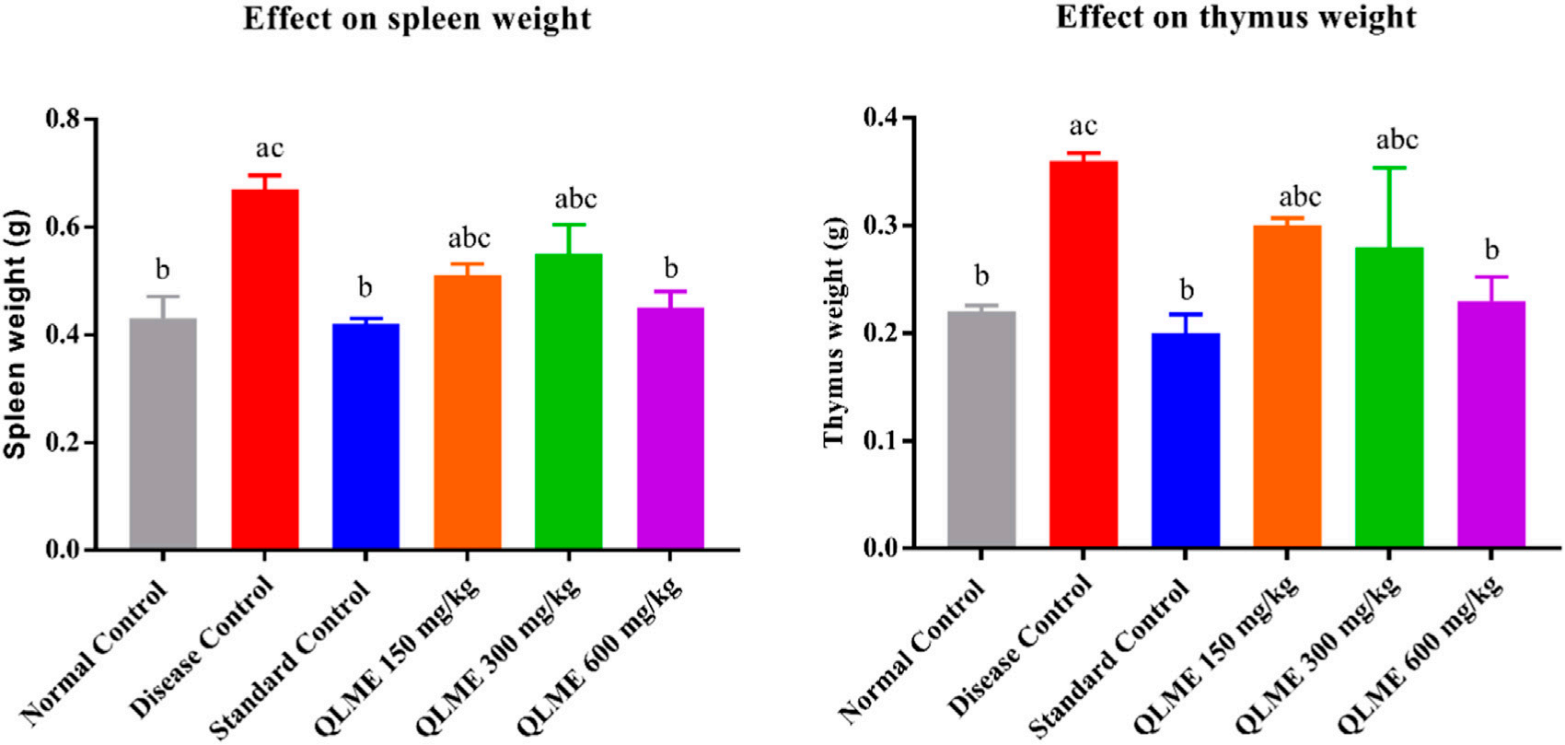
Effects of *Quercus leucotrichophora* extracts on immune organ weight in CFA-induced arthritic rats. Results are expressed as mean ± S.D (n = 6) and one-way ANOVA followed by Tukey’s test. a: normal control; b: disease control; and c: standard control.

The weight of the spleen and thymus increased in DC than in NC. The weight of the spleen and thymus was significantly (*p* < 0.05) restored by QLME (spleen: 0.45 ± 0.03 g; thymus: 0.23 ± 0.02 g) at 600 mg/kg- and MTX (spleen: 0.42 ± 0.01 g; thymus: 0.20 ± 0.02 g)-treated groups in contrast to DC and NC, but the weight of immune organs in the QLME 600 mg/kg-treated group was insignificantly varied from NC and standard control, as shown in Figure 5.

### Inflammatory biomarkers

**FIGURE 6:**
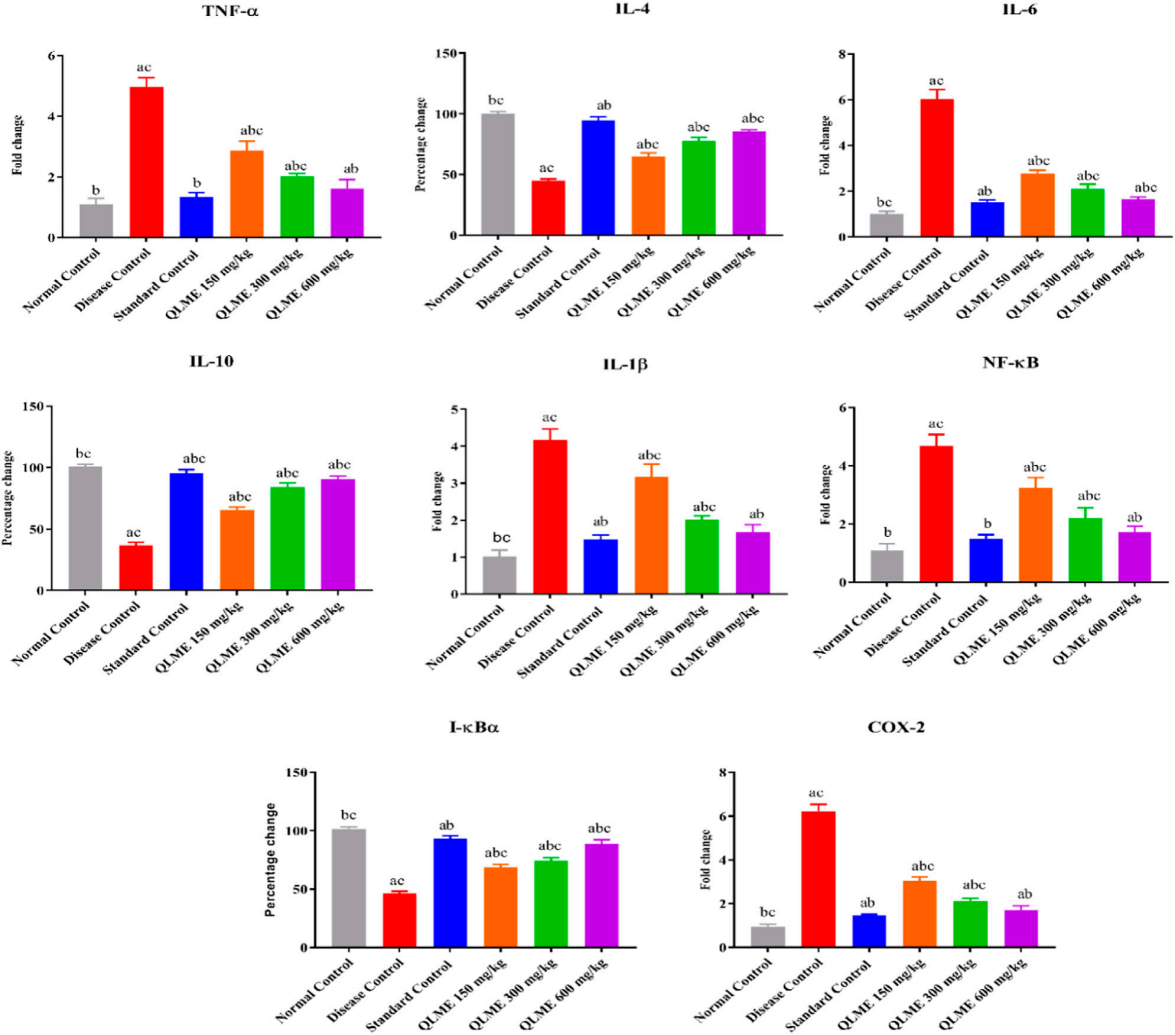
Effects of *Quercus leucotrichophora* extract on mRNA expression of inflammatory biomarkers in arthritic rats. Results are expressed as mean ± S.D (n = 6) and one-way ANOVA followed by Tukey’s test. a: normal control; b: disease control; and c: standard control. IL: interleukin; TNF-α: tumor necrosis factor; COX-2: cyclooxygenase-2. *p* < 0.05 in contrast to DC, NC, and standard control groups.

The expressions of multiple inflammatory biomarkers were quantified by qRT-PCR in all rats after 28 days of CFA inoculation. It was observed that there was a remarkable (*p* < 0.05) downregulation of IL-10 (36.87% ± 2.23%), IL-4 (45.03% ± 1.30%), and I-kBα (46.17% ± 1.86%) along with upregulation of NF-kB (4.67 ± 0.40); COX-2 (6.23 ± 0.30); IL-6 (6.03 ± 0.42); TNF-α (4.97 ± 0.31); and IL-1β (4.17 ± 0.31-fold change) in DC as compared to NC and standard control groups. The level of these biomarkers was restored in all treatment groups in contrast to DC, NC, and standard control groups (Figure 6). However, treatment with MTX (IL-10: 95.67 ± 2.52; IL-4: 94.67 ± 3.01; I-kBα: 93.33% ± 2.52%) and QLME (IL-10: 90.67 ± 2.52; IL-4: 85.33 ± 1.53; I-kBα: 93.33% ± 2.52%) at 600 mg/kg/day had significantly (*p* < 0.0001) increased the expression of these genes in comparison to the DC and NC groups. Moreover, the expressions of IL-6, IL-1β, NF-κB, TNF-α, and COX-2 were significantly reduced by the QLME (NF-kB: 1.71 ± 0.20; COX-2: 1.72 ± 0.19; IL-6: 1.66 ± 0.09; TNF-α: 1.62 ± 0.29; and IL-1β: 1.67 ± 0.20-fold change) 600 mg/kg and MTX (NF-kB: 1.48 ± 0.15; COX-2: 1.45 ± 0.06; IL-6: 1.52 ± 0.09; TNF-α: 1.34 ± 0.14; and IL-1β: 1.47 ± 0.12-fold change)-treated rats in contrast to DC and NC, as given in Figure 6. TNF-α, IL-1β, IL-6, NF-κB, and COX-2 levels in QLME 600 mg/kg were insignificantly varied from those of the standard, while standard control was insignificantly different from NC. IL-4, IL-10, and I-kBα in all the QLME-treated groups were notably (*p* < 0.05–0.0001) varied from NC and standard control.

### Effect on oxidative stress biomarkers

**FIGURE 7:**
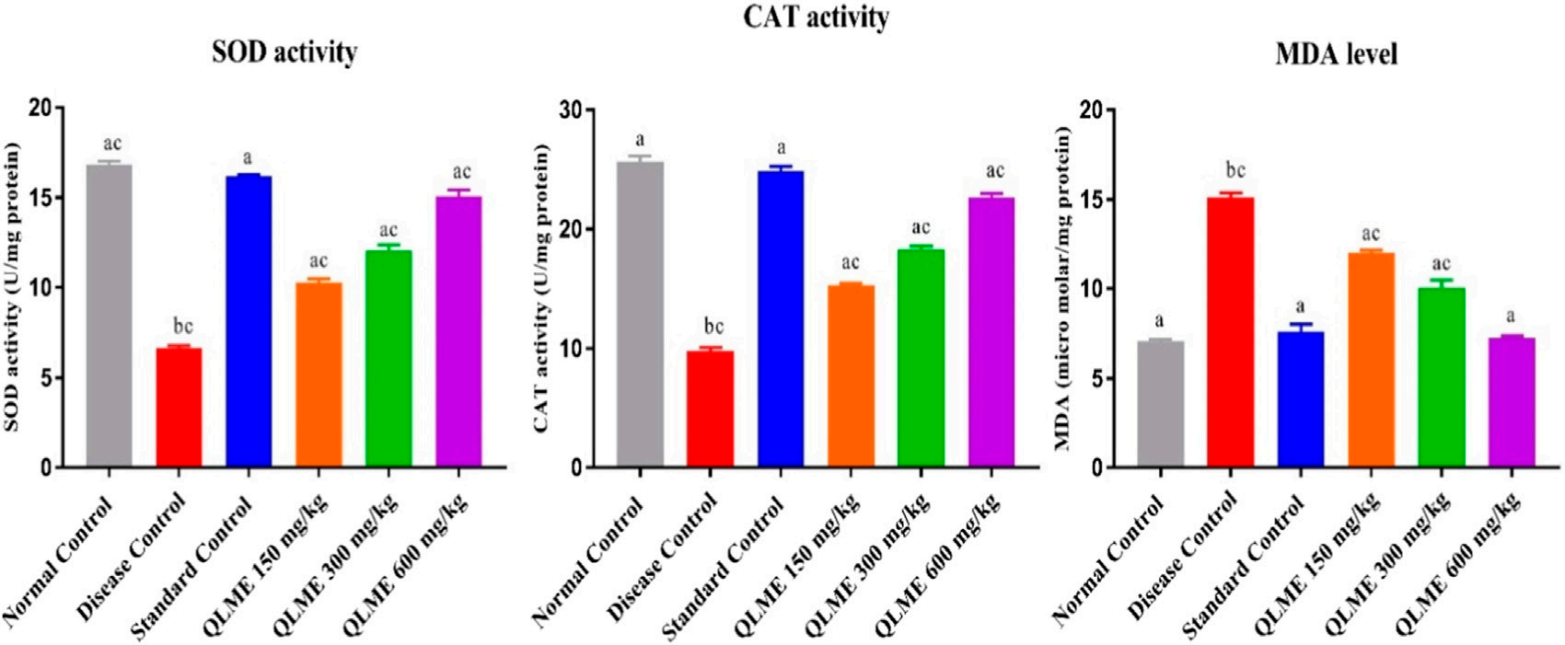
Effects of *Quercus leucotrichophora* extract on oxidative stress biomarkers in CFA-induced arthritic rats. Results are expressed as mean ± S.D (n = 6) and one-way ANOVA followed by Tukey’s test. a: disease control; b: normal control; and c: standard control. *p* < 0.05 in contrast to DC, NC, and standard control.

Oxidative stress was developed after CFA immunization in all rats, which was restored with the extract at all tested dosages and MTX-treated rats (Figure 7). CAT and SOD activities were significantly (*p* < 0.05) lowered in DC in contrast to NC and standard control. There was also an increased level of MDA in LH of DC as compared to NC and standard control. All the treatment groups (MTX and QLME) significantly (*p* < 0.05) improved the activities of CAT and SOD and reduced the level of MDA in arthritic rats as compared to DC and NC. The MDA level and CAT activity in standard control were insignificantly varied from those of NC. The QLME 600 mg/kg (CAT: 22.64 ± 0.35; SOD: 15.08 ± 0.34 U/mg of protein and MDA: 7.26 ± 0.12 µM/mg protein)- and MTX (CAT: 24.89 ± 0.25; SOD: 16.17 ± 0.11 U/mg of protein and MDA: 7.61 ± 0.40 µM/mg protein)-treated groups significantly (*p* < 0.0001) restored the levels of oxidative stress biomarkers in contrast to DC and NC, as shown in Figure 7.

### Effect on joint histopathology

**FIGURE 8:**
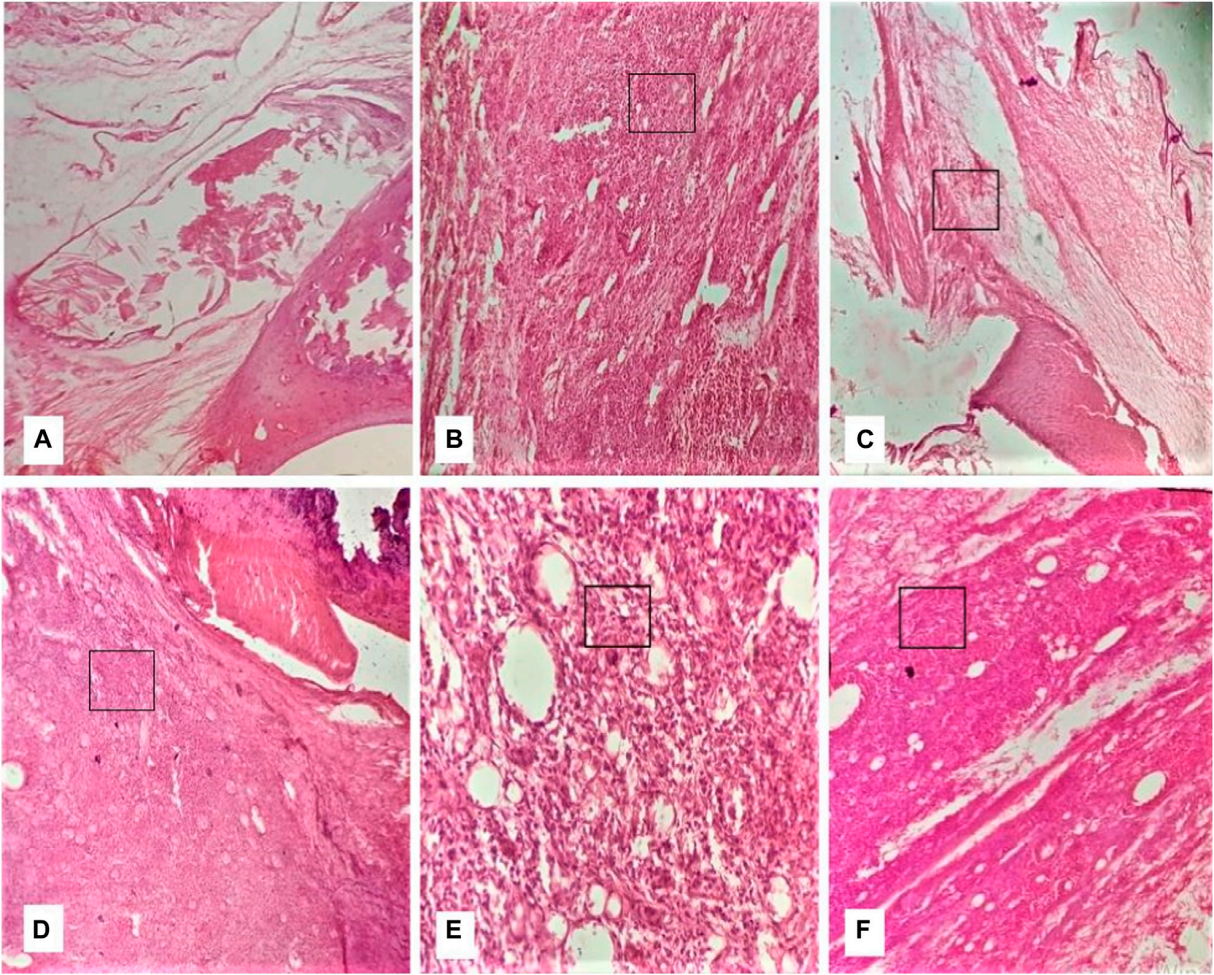
Effect of *Quercus leucotrichophora* on histopathology of CFA-induced arthritic rats. **(A)** normal group, **(B)** diseased group, **(C)** standard group, **(D–F)** QLME at 150, 300, and 600 mg/kg, respectively.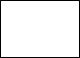 shows inflammation.

At the end of the 28th day, the histopathology of ankle joints showed the formation of pannus, inflammation, bone erosion, and mononuclear cell infiltration in DC (Figure 8). Furthermore, NC rats were devoid of bone erosion and inflammation (Figure 8). The treatment with QLME (150, 300, and 600 mg/kg) and MTX profoundly reduced inflammation, pannus formation, bone erosion, and cellular infiltration in arthritic groups in comparison to DC, as exhibited in Figures 8C–F.

## Discussion

The current study assessed the anti-inflammatory and anti-arthritic potential of QL. The QLME extract had shown notable anti-oxidant and *in vitro* anti-arthritic activities than the QLAQ extract; therefore, it was further evaluated in animals using acute anti-inflammatory tests and CFA-induced arthritis.

Excessive production of free radicals causes oxidative stress, a hallmark of numerous diseases like arthritis, cancer, and diabetes mellitus. Medicinal plants containing anti-oxidants reduce the intensity of disease associated with oxidative stress in humans. The QLME exhibited prominent anti-oxidant activity than QLAQ, probably due to the occurrence of high amounts of TFC and TPC. The flavonoids and phenols having high amounts of hydroxyl groups in plants reduced the free radicals and suppressed oxidative stress as previously described (Arulselvan et al., 2016). The QLME was safe in the acute toxicity study as it did not cause mortality till the 14th day, and LD_50_ was more than 2000 mg/kg.

Phytochemicals like quercetin, chlorogenic, vanillic, gallic, sinapic, and p-coumaric acids protected lysosomal degradation *via* free radical scavenging activity (Oyeleke et al., 2018). Protein denaturation leads to RA by the production of autoantibodies at the target site. It occurred in the presence of strong stimulus like heat, resulting in loss of the protein structure (Akhtar, 2020). Therefore, it can be assumed that the QLME showed the maximum inhibition of protein denaturation and HRBC membrane stabilization than QLAQ due to the presence of higher amounts of flavonoids and phenols as detected by HPLC analysis. It has been reported in previous studies that plants containing phenolic compounds like ferulic, gallic, vanillic, p-coumaric, and sinapic acid and quercetin and catechin have anti-oxidant, anti-inflammatory, and *in vitro* anti-arthritic activities (Roychoudhury et al., 2021). Quercetin inhibited neutrophil infiltration in *in vitro* and *in vivo* studies (Wang et al., 2021).

Xylene and carrageenan are considered significant inducers of inflammation. Xylene is partially involved in substance-P release, causing neurogenic inflammation, leading to release of kinins and histamine with concomitant swelling. The QLME reduced ear inflammation more than QLAQ, and its activity was comparable to that of piroxicam. A similar finding was reported previously by other *Quercus* species (Shabbir et al., 2018). Carrageenan induced inflammation by its actions on the complement system or *via* mediators of inflammation like histamine, prostaglandins (PGs), and 5-HT. In our findings, QLME at 600 mg/kg significantly reduced paw edema by inhibiting the release of inflammatory mediators like PGs, serotonin, and histamine from the activated immune cells (Peerzada et al., 2020).

The CFA was used promisingly to induce polyarthritis in rodents. The CFA suspension contains *Mycobacterium tuberculosis* killed by heat in un-metabolized oils that are favorably used for induction of polyarthritis in rodents that is similar in pathogenesis and symptoms to RA in humans (Saleem et al., 2020c). CFA caused arthritis in two phases. In the primary phase, inflammation occurred with the release of PGs, whereas the secondary immunological phase resulted in the production of auto-antibodies. It leads to the accumulation of mononuclear and polynuclear cells in joints, which results in structural changes in joint and cartilage *via* changes in cytokines (TNF-α, IL-6, and IL-1β), chemokines, tissue destructive enzymes (proteases and lysosomes), and anti-oxidant enzymes. In the current study, CFA immunization caused notable paw edema and weight loss in DC, which were restored with plant extract dose-dependently as evidenced from arthritic indices and histological findings. Weight loss is the prime feature of RA directly related to joint inflammation and might be due to malabsorption of nutrients from the intestine, muscle proteolysis, allodynia, hyperalgesia, and disease distress (Yin et al., 2019; Saleem et al., 2020c). In the current study, CFA-induced weight loss was notably restored with treatment, as evidenced by reduction in paw edema.

TNF-α causes joint destruction and synovial infiltration in RA and causes the formation of other pro-inflammatory cytokines. IL-1β causes morning stiffness and bone erosion in RA. The increased levels of CRP, anemia, and reduced levels of albumin are also due to increased level of IL-6, which leads to myocardial infarction and an extra-articular manifestation of RA (Hashizume and Mihara, 2011). The expressions of pro-inflammatory cytokines (IL-6, IL-1β, NF-κB, COX-2, and TNF-α) were reduced with treatment at all dosage levels, as evidenced by the improvement in blood parameters of arthritic rats also.

IL-4 is involved in Th2 cell production. IL-4 and IL-10 halt pro-inflammatory cytokine release from monocytes and synovial fibroblasts, and their levels were reduced in RA patients, as also evident in DC animals in the current study (Mateen et al., 2016). The levels of anti-inflammatory cytokines were increased in treated rats, as co-evidenced by the reduction of paw inflammation.

As reported in previous studies, quercetin caused inhibition of late-phase inflammation by the inhibition of TNF-α, lipoxygenase, COX-2, and phospholipase A2 (Yin et al., 2019). The QLME at 600 mg/kg/day showed profound anti-arthritic activity comparable to that of the MTX-treated group. The presence of quercetin, sinapic acid, ferulic acid, and other phenolic compounds and flavonoids in QLME might be responsible for its anti-arthritic potential by downregulating pro-inflammatory and upregulating anti-inflammatory cytokines in treated rats (Foyet et al., 2015).

Anemia is the characteristic feature of RA, as reported in diseased rats, which was noticeably restored by QLME at all dosage levels (Saleem et al., 2020c). The systemic biomarkers of arthritis like RF, CRP, ESR, WBCs, and platelets were notably altered in arthritic rats that were restored by treatment (Shabbir et al., 2018). Hyper-functioning of the immune system is recognized from splenomegaly and lymphadenopathy. The QLME notably suppresses the overactive immune system by reducing the weight of immune organ (spleen and thymus), that might be due to the presence of phytochemicals (Saleem et al., 2020c). Enhanced ROS generation occurred because of increased discharge of inflammatory cytokines from activated immune cells like neutrophils and macrophages, ultimately leading to synovium cellular infiltration (Ishibashi, 2013). In the current study, oxidative stress was reduced in treated arthritic rats in contrast to the DC group. Numerous other species of the *Quercus* genus such as *Q. dilatata*, *Q. incana*, *Quercus sideroxyla, Quercus durifolia, Quercus eduardii,* and *Quercus macrocarpa* have exhibited anti-inflammatory, anti-oxidant, and anti-bacterial activities (Moreno-Jimenez et al., 2015; Taib et al., 2020)*;* (Burlacu et al., 2020).

Sinapic acid retains anti-oxidant, anti-inflammatory, anxiolytic, and anti-cancer activities (Bin Jardan et al., 2020). Catechin, a polyphenol, exhibited anti-inflammatory, antioxidant, and anti-hypertensive activities along with various other health benefits (Lee et al., 2020). Sinapic, gallic, vanillic, and ferulic acids, and quercetin and catechin detected in QLME might be responsible for *in vitro* and *in vivo* anti-inflammatory and anti-arthritic potential, as reported in previous studies (Shabbir et al., 2018). Moreover, due to the presence of these phytochemicals in QLME, the cardinal signs of inflammation have also improved in treated animals. Analysis of inflammatory and other molecular biomarkers should be performed by using other techniques, including Western blot.

## Conclusion

It was concluded that QL exhibited *in vitro* anti-oxidant and anti-arthritic activity along with *in vivo* anti-inflammatory and anti-arthritic activity in Wistar rats, which might be due to the presence of ferulic acid, sinapic acid, gallic acid, and catechin and quercetin as detected with HPLC. QLME exhibited anti-arthritic potential by restoring altered blood parameters, pro-, anti-inflammatory, and oxidative stress biomarkers in treated rats. Activity-guided fractionation of *Quercus leucotricophora* should be performed in order to find the most active fraction. There is an immense need for activity-guided fractionation of QLME to isolate anti-arthritic components as well. HPLTC fingerprinting of plant extracts should be performed. Moreover, there should be a detailed toxicity study of QLME to assure its safety for human use.

## Data Availability

The original contributions presented in the study are included in the article/Supplementary Material; further inquiries can be directed to the corresponding authors.
